# Key Messages in an Early Childhood Obesity Prevention Intervention: Are They Recalled and Do They Impact Children’s Behaviour?

**DOI:** 10.3390/ijerph16091550

**Published:** 2019-05-02

**Authors:** Carola Ray, Karen Campbell, Kylie D. Hesketh

**Affiliations:** 1 Folkhälsan Research Center, Topeliuksenkatu 20, 00250 Helsinki, Finland; 2 Department of Public Health, Clinicum, University of Helsinki, 00014 Helsinki, Finland; 3 Department of Food and Environmental Sciences, University of Helsinki, 00014 Helsinki, Finland; 4 Institute for Physical Activity and Nutrition (IPAN), School of Exercise and Nutrition Science, Deakin University, Geelong, VIC 3222, Australia; karen.campbell@deakin.edu.au (K.C.); kylie.hesketh@deakin.edu.au (K.D.H.)

**Keywords:** early childhood, obesity prevention, health messages, intervention, follow-up, food intake, physical activity, screen time, health behaviours

## Abstract

Knowledge of the impact of health messages as an intervention strategy is sparse. The aim of this study was to explore recall and use of health behaviour messages among mothers, and whether recall is associated with child health behaviours. Intervention group data from the 15 months Melbourne Infant Feeding, Activity and Nutrition Trial (InFANT) were used (*n* = 127, children 4 months at commencement). Mothers recalled (unprompted then prompted) at 2 and 3.5 years post-intervention six key messages used in the program, and reported whether they had used them. Children’s food intake was measured by three days of 24-h recall; physical activity by accelerometers; and television viewing by parent report. Unprompted recall ranged between 1–56% across messages and follow-up points, and 37–90% for prompted recall. The most commonly recalled messages “tap into water”, “parents provide, kids decide” and “color every meal with fruit and veg” were also most commonly used. There were few associations between recall and children’s health behaviours. Given the association between recall and reported use, it is important to plan messages so they resonate well with the target group and its needs. Messages should be used as one of multiple strategies within health promotion programs.

## 1. Introduction

Childhood overweight and obesity represent a worldwide health issue [1], as they are connected in the short and long term to children’s physical, psychosocial, and social wellbeing [2,3]. The World Health Organization endorses prevention from the beginning of life, as progression to adiposity begins early, with many children already overweight or obese by the age of two [4]. Parents play a key role in the formation of children’s health behaviours from birth [5]. This provides important rationale for obesity prevention interventions to focus on families with young children.

The understanding of successful parent-focused strategies to prevent childhood obesity is sparse, even though several studies have been conducted [5,6,7,8,9]. Based on previous research, recommendations about how to plan and design family-based childhood obesity prevention studies have been published [10]. Traditionally, most interventions are delivered in-person using written educative materials [5]. The use of messages or slogans to influence or motivate parents to conduct health-promoting practices that will benefit their children have been reported in few studies [11,12]. One study delivering physical activity promoting messages to parents of 9–12 year olds during three years in order to influence parents’ attitudes, beliefs, and support for their children’s physical activity, reported that remembering the messages after the three years of campaign predicted parents’ positive attitudes and beliefs regarding the importance of physical activity [11]. That study also reported that message recall was positively associated with parents spending more time being physically active together with their child [11]. Clearly it is essential for health messages to be remembered or recalled, in ordered to influence the behaviours [13]. It has been argued that health messages are better recalled if they are perceived as fun, positive, related to the receivers daily life, and are delivered at the time a person is needing advice or help [14]. To the best of our knowledge, no studies have reported mothers’ recall and use of messages in promoting health behaviours in younger children, or mothers’ longer term message recall, and its associations with targeted children’s health behaviours.

The Melbourne Infant Feeding, Activity and Nutrition Trial (InFANT) Program is a 15 month early childhood intervention promoting healthy infant lifestyle behaviours (improved diet, physical activity and reduced sedentary behaviours), delivered to first-time mothers. The program utilized six key messages to anchor program content, as one of a number of intervention strategies. In the process evaluation, conducted during the program and 3–5 months post-intervention, mothers perceived many program components as useful and enjoyable, and the key messages were particularly mentioned as sticking in their minds [15]. The aim of this study is to examine the recall of the key messages, and mothers’ use of these, two and 3.5 years post intervention conclusion. Additionally, the study will explore whether mothers’ recall of key messages post-intervention is associated with targeted health behaviours post-intervention among their children (diet, physical activity, and television viewing).

## 2. Materials and Methods

The Melbourne InFANT Program was a cluster-randomized controlled trial run in Melbourne, Australia between 2008 and 2010. The aim of the intervention was to promote positive infant feeding, physical activity and reduced sedentary behaviours (e.g., television viewing) to first-time mothers [16]. There have been two follow-ups; two and 3.5 years post-intervention (2011–2013) [17]. Ethical approval for the Melbourne InFANT Program and for the Melbourne InFANT Program follow-up was provided by the Deakin University Human Research Ethics Committee (ID number: DUHREC 175-2007) and by the Victorian Office for Children (Ref: CDF/07/1138).

### 2.1. Participants

The sample of first-time mothers and their infants were recruited through a two-stage process where the study first randomly selected 14 local government areas in Melbourne, Australia [18]. Thereafter first-time parents’ groups from the local areas were randomly selected for inclusion and randomized at the group level. The first-time parents’ groups are initiated by the universal Maternal and Child Health Service to bring together new parents in local areas for social engagement. The methodology used in the Melbourne InFANT Program has been reported in detail elsewhere [18].

The program recruited 62 groups comprising 542 mother-infant dyads (participation rate 86%). Of those, 271 were randomized to the intervention arm and are included in this study. The control group were excluded from this study as they were not exposed to the intervention messages. Infants were about four months of age when the program started and the program lasted 15 months (retention rate 91%). The first post-intervention follow-up occurred two years after the conclusion of the intervention when children were aged 3.5 years, and the second 3.5 years after conclusion of the intervention when children were aged five years. A total of 187 (69% retention rate) and 179 (66% retention rate) mother-child dyads took part in the two and 3.5 year follow ups, respectively.

### 2.2. The Intervention

The 15 month intervention included six by 1.5 h sessions. The Melbourne InFANT program used an anticipatory guidance framework to support mothers to apply practices that promote healthy energy-balance behaviours among their children [18]. Each session was led by an experienced dietitian and the sessions were focused on parental knowledge, skills, and social support promoting improved infant feeding, diet, physical activity and reduced television viewing.

The program had six key messages that provided the cornerstone of the messaging throughout the intervention. The key messages included one established message ‘parents provide kids decide’ [19], one used by the state department of health in Victoria ‘tap into water’, and four developed by the research team to provide focus on key outcome behaviours. In the development phase there was a small consumer trial (*n* = 10) testing the target audience’s responses to the key messages and messages were modified accordingly. The key messages were written so that they were applicable to all childhood stages and they remained the same throughout the intervention. They were repeated frequently at each group session, in all the materials and some were printed on participant gifts (shopping bags and lunch boxes). The key messages where operationalized so that the topics discussed during the sessions were in line with the key messages, but adapted to the upcoming age status of the children. Certain messages were highlighted more strongly at specific sessions. Each key message focused on specific parenting approaches (Table 1).

### 2.3. Follow-up Measurements

Families who participated in the Melbourne InFANT program were invited to participate in the follow-up studies [17]. New written consent was given by participants. Questionnaires were sent to participants by mail and collected by research staff during scheduled home visits. At the home visit, children were fitted with accelerometers to wear during waking hours for one week, after which the monitors were collected by research staff or posted back. After the home visits three unscheduled days of 24 h dietary recalls were conducted via telephone interviews. All data collection and measures were assessed in the same way at both post-intervention follow-ups.

Prior to commencement of the first of the three dietary recall telephone interviews (described below), mothers who had been in the intervention arm were asked the following: “Throughout the InFANT Program there were some key messages discussed. Can you remember any of these key messages?” If a message was recalled (correctly or incorrectly) the interviewer asked “Are there any other key messages you can recall from the program?”. This continued until the participant recalled no further messages. If the participant did not recall particular key messages the interviewer then prompted recall by asking them: “Do you remember the message…?”. This was repeated for each message that had not been recalled. Based on responses to these questions, a derived variable was created for each key message with three response categories: (1) the key message was recalled unprompted, (2) the key message was recalled when prompted, and (3) the key message was not recalled even if prompted. For the statistical analyses two variables based on the recall answers were formed. Firstly, a dichotomous variable for each specific key message indicating whether the key message was recalled (0 = not recalled or recalled when prompted, 1 = recalled unprompted). The division into two groups was informed by the distribution of the answers to the question regarding recall of the key messages. The distribution of responses to the recall of key messages was skewed informing the decision to create a dichotomous variable. Secondly, a continuous summary variable was formed; not recalling any of the key messages even if prompted gave the lowest score, 0, and recalling all key messages unprompted gave the highest possible score of 12 (i.e., describe derivation). Hereafter this variable was named total message recall.

Mothers were asked an additional set of questions about the use of the key messages: “Have you used the following messages from the Melbourne InFANT Program in the past 2 years/3.5 years since the program finished?”. The six key messages were listed one by one and the participant answered yes or no for each message.

Children’s physical activity was objectively measured by a hip worn ActiGraph GT1M accelerometer (ActiGraph, Pensacola, FL, USA) which was worn for eight consecutive days during waking hours. Data were collected in 15 s epochs and those with 4 or more day’s data with a minimum of 7.4 h monitoring per day, were included in analyses [20]. Time (average minutes per day) that the child spent in light-, moderate-, and vigorous-intensity physical activity (LMVPA) was used in analyses, and the cut off for LMVPA was >100 counts per minute [21].

Sedentary behaviour was assessed in this study as TV viewing time. Mothers completed a questionnaire that assessed average TV viewing time on weekdays and weekend days [22]. Average daily TV viewing time was calculated by summing average weekday (×5) and weekend day (×2) viewing time and dividing by seven.

The dietary intake of children, comprising all foods and drinks consumed, were assessed by 24 h recall telephone interview. Mothers were telephoned unscheduled on three non-consecutive days to report on their child’s intake the day before [16]. Calls were administrated so that the recall captured one weekend day and two weekdays and the procedure for all three calls was standardized. The dietary analysis program FoodWorks was used to analyse dietary data (FoodWorks, Dietary Analysis Program 3 ed.; 2006, Xyris Software, Brisbane, Australia). The following variables were generated and used in the current analyses: Average daily intake (g/day) of fruits (fruit juices excluded), vegetables (potatoes excluded), non-core sweet foods (such as sweets, chocolate, cakes), and water. A high proportion of children had no daily intake of non-core savory foods (such as crisps, savory biscuits, appr. 28%) and non-core drinks (such as sweetened fruit juices, soft drinks, approximately 42%) at two year follow-up. These variables were used as dichotomised variables in the current analyses.

### 2.4. Statistical Analyses

Means and standard deviations were calculated for background factors and for children’s dietary intake, time spent in physical activity and use of screens. To examine whether there were any differences in distribution in who recalled the specific key messages or used the key messages at follow-ups 2 and 3.5 years, paired sample t-test was used.

Three association analyses were undertaken to assess the study questions. Firstly, associations between each separate key message recall and the use of the key message, both cross-sectional and longitudinally were examined using multiple multilevel logistic regression analyses. Secondly, associations between the total key messages recalled and the children’s health behaviours cross-sectional and longitudinally were examined using separate multilevel linear models. Thirdly, separate multilevel linear and logistic models in cross-sectional and longitudinal analyses were used to assess whether recall of a specific key message was associated with a health behaviour related to that specific key message.

The clustering by first-time parents’ group was accounted for in analyses, and adjustments were made for the highest education level of mothers. Mother’s highest education was categorized into three groups; high school or lower education, trade or certificate, and university degree or higher. When examining children’s physical activity the analyses were, in addition, adjusted for accelerometer wear time. SPSS version 24 (IBM Corp., Armonk, NY, USA) was used for the analysis, except the multilevel logistic regression analyses where Stata 13.0 (Stata Statistical Software, Stata Corp., College Station, TX, USA) was used. The statistical significance level was set at *p* < 0.05.

## 3. Results

The mean age of mothers in the beginning of the Melbourne InFANT program was 32.6 years and nearly all mothers, 95.1% (*n* = 117), were first time mothers. At the two year follow-up the vast majority lived in a registered marriage or in de facto relationship (98%), 20.3% with a high school or lower education, 26.7% with trade/certificate, and 53.3% with an university degree. The distribution in the educational level among participating mothers at 3.5 year follow-up remained the same. The mean age of children at the 2 year follow-up was 3.6 years, and at the 3.5 year follow-up 5.1 years. There were slightly more girls than boys participating (54% and 57% girls at respective follow-ups).

There was variation in how well key messages were recalled and used (Table 2). “Eat together play together” was recalled unprompted by few mothers (1% and 2%), whereas nearly all recalled it when prompted. Another key message having a low unprompted recall was “off and running”, with 5% and 10% respectively recalling this message at the two follow-ups. “Off and running” was the message least often reported by mothers to have been used at both follow-ups (29.9%). The message “tap into water” was spontaneously recalled by the highest proportion of mothers at both follow-ups (42% and 56%). More than a third of mothers recalled unprompted the messages “parents provide kids decide” and “color every meal with fruit and veg”. Virtually all mothers recalled these three messages when prompted. These were also the messages most often reported by mothers to have been used.

The proportion of recalls differed at the 2 and 3.5 year follow-up for some of the key messages (Table 2). A significantly higher proportion of mothers recalled “tap into water” (55.9% vs. 41.5%; *p* = 0.002), and “off and running” (10.5% vs. 4.9%; *p* = 0.001)) unprompted at 3.5 year follow-up than at 2 year follow-up. Simultaneously, a higher proportion of mothers did not recall “tap into water” at all at 3.5 years follow-up compared to the 2 year follow up (1.6% vs. 4.2%; *p* = 0.001). The key message “off and running” went in the opposite direction, at 2 year follow-up 58% did not recall the key message whereas at 3.5 year follow-up 49% did not recall the message (*p* = 0.001). For all other messages recall was similar at the two follow-ups. The proportion of mothers who reported using “parents provide kids decide” decreased from 2 year follow-up to 3.5 year follow-up (Table 2, 70.9% to 52.8%; *p* = 0.007).

Mothers who recalled unprompted the key messages “parents provide, kids decide” and “off and running” at 2 year follow-up had higher odds of reporting that they were using that specific key message at the same time point than did those mothers who recalled it only when prompted or not at all (Table 3). Similarly, there were cross-sectional associations at 3.5 year follow-up between unprompted recall of the message and use for each of “parents provide kids decide”, “off and running”, and “tap into water”. Recalling a key message unprompted at 2 year follow-up did not predict later use of that specific key message at 3.5 year follow-up.

Associations between total message recall and children’s health behaviours are reported in Table 4. Mothers who recalled more of the key messages at 3.5 year follow-up had children who consumed less non-core sweets at 3.5 year follow-up. No other cross sectional or longitudinal associations were seen between the total message recall variable and child health behaviours.

Finally, associations between recall of specific key messages and the corresponding health behaviour(s) were tested. Testing the separate associations generated 102 tests of which four showed significant associations. Unprompted recall at 2 year follow-up of “eat together play together” positively predicted children’s water intake at 3.5 year follow-up (unstandardized beta 885.55, 95% CI 267.71 to 1503.40, *p* = 0.005). Unprompted recall of “parents provide kids decide” at 3.5 year follow-up demonstrated a negative cross-sectional association with children’s fruit intake (unstandardized beta −40.68, 95% CI −79.86 to −1.50, *p* = 0.04). Unprompted recall of “snack on fruit and vegetables” at 2 year positively predicted children’s non-core sweet intake at 3.5 year follow-up (unstandardized beta 12.07, 95% CI 0.63 to 23.52, *p* = 0.04). Recalling the key message “color every meal with fruit and vegetables” unprompted at 3.5 year follow-up was cross-sectionally associated with children’s fruit intake (unstandardized beta 41.74, 95% CI 4.65 to 78.83, *p* = 0.03).

## 4. Discussion

The aim of this study was to examine whether mothers participating in the Melbourne InFANT Program recalled and used the intervention key messages 2 and 3.5 years post-intervention. Additionally, the study explored whether recall of messages was associated with targeted children’s health behaviours. While the use of messages in health promotion is seen as important [12], to the best of our knowledge no other study has reported the post-intervention recall or use of key messages.

The recall of the six messages used in the Melbourne InFANT program varied substantially. The most recalled key message at both 2 and 3.5 year follow-up was “tap into water”. One plausible explanation for the high recall of this message may be that the message was used by the Victoria state department of health in a social marketing campaign during the same years that the Melbourne InFANT program was conducted. Thus mothers were likely to have additional exposure to the message, beyond that within the program. It is has been proposed, and seems likely, that a national campaign can bolster local health promotion work [23]. Another explanation for the higher recall of this message could be its novelty. While many nutrition projects have promoted fruit and vegetable intake, promoting water intake for young children may have seemed novel for the target group. Consistent with this notion of novelty as a facilitator of recall the key message “parents provide, kids decide”, also commonly recalled, was noted as a novel message in earlier qualitative interviews [15,24]. All messages in the Melbourne InFANT program were designed to be short and descriptive and focus tested for relevance. The novelty, the relevance for the receiver, the short and descriptive form as appealing to the audience, are factors that have been proposed to be successful strategies for health messages [12,14].

The key message “off and running” had quite low recall. This message related to increasing physical activity and reducing screen time. At commencement of the Melbourne InFANT program children were babies and remained largely non-mobile for much of the intervention period. Diet may have been a more relevant and tangible theme for mothers than physical activity or television viewing at this age, which might have affected recall. While mothers often find early feeding a challenging time [25], physical activity is typically less of a concern with many mothers perceiving they do not need to actively encourage their young child to be active [26,27]. It could also be that this key message was not as obvious and well understood as the other messages. However, mothers who spontaneously recalled this message did report using it suggesting that it was a useful tool for those the message resonated with.

Reported use of the key messages recalled unprompted varied substantially, (30–70%), but was relatively high when considered in relation to unprompted recall which was as low as 1%. That the use of some of the key messages was much higher than the recall of that message could be due to using strategies which the messages have promoted within the program, without actually recalling the message itself. This could also explain why association between recall and use of key messages were found for only some of the key messages. In the process evaluation of the Melbourne InFANT program mothers reported that applying the key messages into practical situations facilitated their daily lives, so it could be that the use of strategies had become a behavior [15]. The engagement with the key messages suggests the practical strategies might be important mediators of uptake of the messages and its applications.

With very few exceptions, associations were generally not shown between recall of the key messages and children’s health behaviours. The link between what mothers recalled and children’s health behaviours might have been too distant to show associations. It is not expected that messages would have a direct effect on child behaviour so it may be that it is more appropriate to consider impact on the mediating factors such as parenting practices. A study among 9–12 year-old’s parents which also used key messages, reported that parents who recalled the physical activity focused key messages better had more physical activity days with their children [11]. Further, assessment of both recall and behaviours occurred in our study two and 3.5 years after conclusion of the intervention. It is possible we may have detected greater association if these had been assessed during or immediately after the intervention when both recall and use may have been stronger.

This study found a few associations between recalled key messages and children’s health behaviours. Even if the key messages were quite universal and applicable to all age stages, mothers might not have been able to apply them as children grew older. Another explanation might be that recalling key messages might influence some practices at home i.e., the availability or accessibility of fruit and vegetables. As these factors were not measured, it is not possible to know if the intervention had some other effects than those on than children’s health behaviours. The use of alternative outcomes to the behavioural outcomes in nutrition programs has been proposed previously in order to get a broader understanding of the effects of the intervention [28].

This study has some notable strengths. In involves long term follow-up which is often cited as lacking in the evidence base [6,29]. The methodology of evaluating message recall by telephone interviews enabled knowledge about recalls of key messages unprompted, and further investigation of prompted recall, which is not possible by using questionnaires. This methodology also ensured true recall. As interviews were not prescheduled mothers were not prepared for the call and thus unable to refer to intervention materials to find key messages during the interview.

A limitation of the study is the participant attrition with just two thirds of the intervention participants retained in the follow up phase. It is possible that those retained may have been more engaged in the study and thus more likely to recall messages. However, given the variability in recall this does not appear to have had a major impact. Just over 50% of the participating mothers had a university degree, which could indicate selection bias. However, 46% of Australian women aged 25–40 have a university degree (Australian Bureau of Statistics 62,270 Education and Work, Australia, May 2018), so the participants are broadly reflective of the population.

Further, as the target sample was only the intervention arm of the original trial, sample size from the original study was halved which may have impacted power to detect associations. However this was necessary given the aim of the study as only intervention participants were exposed to the key messages. As recall of the key messages at conclusion of the Melbourne INFANT program was not measured, it was not possible to assess salience of the messages during the program, or how this may have impacted longer term recall at follow-up. The key messages were only one of many strategies utilized to promote children’s health behaviours in the Melbourne InFANT program and it is not possible to fully disentangle the impact of the messages, independent of the other strategies. As the Melbourne InFANT program showed positive impact on a number of child health behaviours [16], which was not reflected in this study, it is likely that the combined effect of the strategies used in the intervention was more important than the impact of any one strategy alone.

## 5. Conclusions

This study demonstrates that key messages used in a health promotion program were generally recalled well by mothers 2 and 3.5 year post-intervention. Given some messages were recalled better than others, it is likely that particular messages had greater salience with the target population and suggests the importance of developing highly relevant messages in consultation with the target group. While there was not a strong link between message recall and target child behaviours, it is important to note that the key messages were only one of a suite of strategies used within the program and it is likely that different strategies impacted differentially on individual participants. This highlights the need for programs to use a variety of strategies to reinforce the aims of the intervention.

## Figures and Tables

**Table 1:** The six key messages in the Melbourne Infant Feeding, Activity and Nutrition Trial (InFANT) program and the parenting approaches behind the messages.

Key Message	Parenting Approach
Eat Together, Play Together	Modelling
Parents Provide, Kids Decide	Division of responsibility
Snack on Fruit and Veg	Food purchase, preparation, availability and accessibility, modelling
Colour Every Meal with Fruit and Veg	Food purchase, preparation, availability and accessibility, modelling
Tap into Water	Drink availability and accessibility, modelling
Off and Running	Substitution of screen time for physical activity availability, modelling

**Table 2:** Proportion of mothers recalling and using key messages at 2 year and 3.5 year follow-ups ^1^ (children aged 3.5 and 5 years).

Recalling Key Messages	2 Year Follow-up	3.5 Year Follow-up	*t* (*p*-Value)
	% (*n* = 123)	% (*n* = 116)	
Eat together Play together			−0.53 (0.595)
Recalled unprompted	0.8	1.7	
Recalled when prompted	88.6	89.8	
Did not recall	10.6	8.5	
Parents provide Kids decide			−1.06 (0.291)
Recalled unprompted	35	33.9	
Recalled when prompted	56.9	58.5	
Did not recall	8.1	7.6	
Tap into water			−3.18 (0.002)
Recalled unprompted	41.5	55.9	
Recalled when prompted	56.9	39.8	
Did not recall	1.6	4.2	
Snack on fruit and veg			−1.38 (0.171)
Recalled unprompted	13	11.9	
Recalled when prompted	66.7	75.4	
Did not recall	20.3	12.7	
Colour every meal with fruit and veg			−1.97 (0.051)
Recalled unprompted	35.8	41.9	
Recalled when prompted	61.8	56.4	
Did not recall	2.4	1.7	
Off and running			−3.30 (0.001)
Recalled unprompted	4.9	10.2	
Recalled when prompted	36.6	40.7	
Did not recalled	58.5	49.2	
% using key messages	% (*n* = 127)	% (*n* = 125)	
Eat together Play together	53.5	40.2	1.87 (0.064)
Parents provide Kids decide	70.9	52.8	2.73 (0.007)
Tap into water	71.7	65.4	1.18 (0.238)
Snack on fruit and veg	48.8	44.9	0.47 (0.641)
Colour every meal with fruit and veg	68.5	65.4	0.47 (0.641)
Off and running	29.9	29.9	−0.29 (0.769)

^1^ Paired sample T-test comparing the two year versus 3.5 year responses in each category.

**Table 3:** Cross-sectional and longitudinal associations between recalling specific key messages ^1^ and the reported use of the key message.

Key Message	Recall and Use at 2 Year Follow-up	Recall and Use at 3.5 Year Follow-up	Recall at 2 Year Follow-up and Use at 3.5 Year Follow-up
	OR (95% CI)	OR (95% CI)	OR (95% CI)
Eat together play together	1.08 (0.84, 1.38)	1.22 (0.93, 1.60)	1.06 (0.83, 1.34)
Parents provide kids decide	1.40 (1.04, 1.87) *	1.36 (1.02, 1.82) *	1.10 (0.88, 1.38)
Tap into water	1.15 (0.91, 1.45)	1.50 (1.03, 2.18) *	1.18 (0.90, 1.55)
Snack on fruit and veg	1.22 (0.98, 1.53)	1.11 (0.88, 1.40)	1.11 (0.90, 1.38)
Colour every meal with fruit and veg	1.18 (0.86, 1.61)	1.25 (0.98, 1.60)	1.05 (0.80, 1.38)
Off and running	1.42 (1.05, 1.90) *	1.38 (1.02, 1.86) *	1.10 (0.82, 1.47)

* *p* < 0.05. Separate multilevel logistic regression models, odds ratios (OR), and 95% confidence intervals (95% CI). All analyses adjusted for clustering of mothers group and the highest education of mothers. ^1^ Independent variable, the recall, was categorized into two categories; not recalling, or recalled when prompted is category 0, and unprompted recall is categorized as 1.

**Table 4:** Cross sectional and longitudinal associations between total message recall (range 0–12) and children’s health behaviours.

Children’s Health Behaviour	2 Year Follow-up	3.5 Year Follow-up	2 Year Follow-up Recall Predicting Health Behaviour at 3.5 Year Follow-up
	Unstandardized B (95% CI)	Unstandardized B (95% CI)	Unstandardized B (95% CI)
Fruit intake (g/day)	2.93 (−10.44, 16.30)	0.023 (−11.89, 11.93)	3.48 (−7.93, 14.90)
Vegetable intake (g/day)	−1.27 (−9.52, 6.98)	5.09 (−5.11, 15.29)	2.48 (−6.81, 11.76)
Water intake (g/day)	23.43 (−13.76, 60.63)	6.52 (−32.41, 45.46)	11.86 (−26.02, 49.74)
Non core sweets (g/day)	−1.29 (−3.42, 0.83)	−3.28 (−6.32, −0.25) *	−1.60 (−3.98, 0.77)
TV viewing time (min/day)	−3.36 (−16.87, 10.15)	−6.65 (−13.91, 0.62)	−5.27 (−12.38, 1.84)
Physical activity (min/day)	2.35 (−31.32, 9.04)	3.82 (−3.27, 10.91)	−4.14 (−11.54, 3.25)
	OR (95% CI)	OR (95% CI)	OR (95% CI)
Non core drinks (no intake vs. some intake/day)	1.00 (0.76, 1.30)	1.06 (0.83, 1.34)	1.00 (0.79, 1.27)
Non core savory (no intake vs. some intake/day)	1.12 (0.87, 1.44)	1.02 (0.69, 1.50)	1.26 (0.92, 1.73)

* *p* < 0.05. Separate multilevel linear and logistic regression models, unstandardized beta, odds ratios (OR), and 95% confidence intervals (95% CI). All models adjusted for clustering of mothers group and mother’s highest level of education, and in the physical activity model for the accelerometer wear time.
